# Maternal Risk Factors Associated with Limb Reduction Defects: Data from the Polish Registry of Congenital Malformations (PRCM)

**DOI:** 10.3390/children8020138

**Published:** 2021-02-12

**Authors:** Anna Materna-Kiryluk, Katarzyna Wisniewska, Barbara Wieckowska, Jolanta Wierzba, Anna Jazdzewska, Beata Jaroszewska-Swiatek, Kinga Skotnicka, Anna Latos-Bielenska

**Affiliations:** 1 Polish Registry of Congenital Malformations, Chair and Department of Medical Genetics, University of Medical Sciences, 61-701 Poznan, Poland; kinga.skotnicka17@gmail.com (K.S.); alatos@ump.edu.pl (A.L.-B.); 2 Epidemiology Unit, Department of Preventive Medicine Poznan, University of Medical Sciences, 61-701 Poznan, Poland; kwisniewska@ump.edu.pl; 3 Department of Computer Science and Statistics, Poznan University of Medical Sciences, 61-701 Poznan, Poland; basia@ump.edu.pl; 4 Department of Internal and Pediatric Nursing, Medical University of Gdansk, 80-952 Gdansk, Poland; jolanta.wierzba@gumed.edu.pl; 5 Surgery and Burn Treatment Unit, Specialist Mother and Child Healthcare Centre in Poznan, 61-825 Poznan, Poland; ajazdz@o2.pl; 6 Department of Clinical Pediatrics, University of Warmia and Mazury in Olsztyn, Children’s Hospital in Olsztyn, 10-561 Olsztyn, Poland; swiatekfam@poczta.onet.pl

**Keywords:** congenital abnormalities, limb deformities, congenital, risk factors

## Abstract

Data from the Polish Registry of Congenital Malformations (PRCM) suggest that the prevalence of limb reduction defects (LRDs) in some Polish regions is significantly higher in comparison to that reported in the European Surveillance of Congenital Anomalies (EUROCAT) registry, but specific risk factors are still unknown. The objectives of this study were two-fold: to detect risk factors linked to isolated LRDs among Polish natives and to search for geospatial clusters of isolated LRDs to identify high-risk areas across the country. Among the 2,939,001 births accounted for in the PRCM, we determined that there were 852 children with distinct LRDs. Our data demonstrate that lower birth weight, prematurity, and maternal smoking history are strongly associated with isolated LRDs. Furthermore, our investigation pointed to various additional risk factors for isolated LRDs, including paternal education, gestational hypertension, upper respiratory tract infections, and exposure to anti-inflammatory drugs in the first trimester of pregnancy. We did not recognize statistically significant spatial or spatiotemporal clusters over the area of Poland using Kulldorff’s scan. Our study strengthens the hypothesis that maternal factors have an integral role in the etiology of isolated LRDs.

## 1. Introduction

Following the Thalidomide tragedy in the 1960s, the prevalence of limb reduction defects (LRDs) became one of the key indicators of teratogenic effects of exogenous factors in humans [1,2]. To date, several risk factors associated with LRDs have been identified. Young maternal age (under 25 years old) has been recognized as one of the factors influencing LRD [3,4]. Obesity and vitamin deficiencies (e.g., riboflavin) during pregnancy increase the fetal risk of LRDs [5,6]. Moreover, certain illnesses, injuries, and exposures to certain medications and chemical substances in the first trimester have been recognized as potential risk factors [3,4,5,6,7,8,9,10,11,12]. Nevertheless, the precise etiology of most LRDs is not well understood. Accordingly, there is no effective way to prevent these types of defects.

A EUROCAT study that included 25 registries (without Poland) from the years 1980–2012 showed a reasonably consistent prevalence of LRDs across Europe after the exclusion of chromosomal aberrations and multiple-defects, and from 2004, a decreasing trend in prevalence was observed [13].

Data from the Polish Registry of Congenital Malformations (PRCM) suggest that the prevalence of isolated LRDs in some Polish regions is significantly higher in comparison to that reported in the EUROCAT registry [14,15,16,17] and other registers, such as the Alberta Congenital Anomalies Surveillance System (ACASS) [18].

Because published EUROCAT data exclude only chromosomal aberrations, a comparison of the prevalence of isolated LRDs between Polish and other European populations has been difficult. In general, however, among all children with LRDs reported to the PRCM in the years 1998–2010, there were less chromosomal aberrations and multiple-defects as compared to the EUROCAT registries (~30% versus ~50%). In aggregate, these data suggest a considerably higher overall prevalence of isolated LRDs in some regions of Poland [14].

Despite its high prevalence, specific risk factors for LRDs in the Polish population remain unknown. The literature suggests the possibility of specific environmental factors contributing to the risk of LRDs, for example, contaminated drinking water [19], justifying further research aimed at identifying potentially preventable environmental exposures.

Population registries of congenital malformations, such as the PRCM, may help to identify specific groups of children at increased risk. The case characteristics can then be used to identify etiologic factors and potentially employ targeted prophylactic strategies.

To date, there are no published data on isolated LRDs, their risk factors, or their geospatial distribution in Poland.

The objectives of this paper are two-fold: first, we aim to study the characteristics of LRD cases and detect specific maternal factors associated with isolated LRDs. Second, we aim to conduct a search for geospatial clusters of isolated LRDs to potentially identify high-risk areas across the country.

## 2. Materials and Methods

The data analyzed in this study were collected as part of the PRCM registry, a member of the EUROCAT network of population-based registries for the epidemiological surveillance of congenital anomalies. The PRCM is a population-based registry. It records data related to all births from mothers residing within the area covered by the Registry, irrespective of where the birth takes place. The main source of information is a registration form completed by the physician diagnosing the anomaly. Electronic reporting has also been implemented since 2004. The registration form is filled out by the physician diagnosing the anomaly. For each child with a congenital anomaly, a detailed description of the congenital malformations is recorded. Other data include the date of birth, birth order, birth weight, gestational age at delivery, the child’s age (or gestational age) at diagnosis, parental age, education and consanguinity, course of pregnancy, risk factors before and during pregnancy (including the mother’s diseases, medications, and addictions), prenatal diagnosis, and family history, including previous pregnancies. Data about maternal medications and diseases in the first trimester of pregnancy were also collected. The information was entered into the Registry database. The coding of malformations was based on the International Statistical Classification of Diseases and Related Health Problems, 10th revision [20]. The coding of malformations was carried out using the EUROCAT guidelines as delineated by the Committee on Classification and Coding of Malformations [21]. Two experienced clinical geneticists coded the malformations. Along with the methods for gathering and saving information about congenital malformations, the organizational structure of the PRCM has previously been described in detail [22]. The evaluation was conducted from 1998 to 2010 in a population that consisted of 2,939,001 births in the region of 11 Polish voivodeships (from 16 covered by the PRCM), which amounted to 59% of all births in the Polish population in those years. The analysis included 11 Polish voivodeships: from 1998—Dolnośląskie, Kujawsko-Pomorskie, Lubuskie, Opolskie, Pomorskie, Wielkopolskie, Warmińsko-Mazurskie and Zachodniopomorskie, from 2001—Śląskie, and from 2002—Lubelskie and Podkarpackie. In the calculation of the overall prevalence of LRDs, we included syndromic, multiple, and isolated cases.

Our detailed analysis only included all live births registered in the PRCM in which isolated LRDs (ICD-10: Q71–Q73) were confirmed from the time of birth to 2 years of age. A baby with an isolated LRD could have defects in one or more extremities, including the arms and legs. The exclusion criteria consisted of malformations of suggested genetic etiology, such as familial cases of LRDs and bilateral LRDs (left and right), known chromosomal aberrations, all known syndromes, and multiple birth defects presenting LRDs. Amniotic bands syndrome was also excluded from the analysis. After applying the above inclusion and exclusion criteria, 852 LRD cases advanced to the final analysis. The information on the number of births from 1998 through 2010 in the studied region came from the Regional Data Bank of the Central Statistical Office [23].

A total of 12,002 healthy controls reported to the registry were matched according to birth year and residential area. However, among the controls, many mothers were younger and had a higher education level compared to the general Polish population. In order to assure that the control group was more representative, we next sub-selected a group of controls that best matched the characteristics of the general Polish population based on data from the Polish Central Statistical Office [23]. To assure a balanced analysis, the sample size of the control group was set to be equal to the size of the case group. Control selections were implemented via stratified sampling from the group of recruited control subjects, taking into account the following strata: maternal age, education status, birth weight, and gestational age of the children. Within each stratum, random sampling was done using the proportionate allocation strategy so that in each of the strata a sampling fraction was proportional to that of the total population. Following this approach, a total of 852 controls were chosen throughout all strata.

The effect of examined factors on the risk of malformations was tested by applying logistic regression models in two stages. First, the crude effects were estimated individually.

Second, we built a multivariable model based on the variables that were significant in univariate analyses. These variables included gestational age, birthweight, gravidity, and maternal tobacco use during pregnancy. Maternal and paternal education were not used for adjustments in any of the regression models because of their significant collinearity with the variables reflecting health-related behaviors, such as tobacco use during pregnancy [24].

In the geospatial analyses, we defined a cluster of congenital malformations as an accumulation of cases in time and/or space that was above the expected average. The analysis of geospatial clusters was conducted on gminas (administrative subregions of maternal residence that were used as units in the geospatial analysis). Among 852 cases, 842 had full information about resident places (gminas); thus, 10 cases with missing data were excluded from this analysis. The search for geospatial clusters was conducted using the Kulldorff’s scan statistic. The statistical significance of the cluster was tested with the likelihood ratio test. The null distribution of the test statistic was estimated with the use of the Monte Carlo method and an empirical *p*-value was derived [25]. The power of the Kulldorff’s statistic increases with the increased relative risk of LRDs for cluster inhabitants. Under the assumption that any potential cluster size cannot exceed 10% of the examined area, our detection power ranged from 13.3% to 98.2% for RR of 2.0 to 4.0, respectively. Statistical calculations were prepared with the use of the PQStat program (1.6.2). The SatScan program (9.4) was used to search for clusters. *p*-Values < 0.05 were considered statistically significant.

## 3. Results

Table 1 presents the number of children and the prevalence of LRDs in the analyzed area. The total prevalence of LRDs was estimated at 5.9 per 10,000 births and ranged between voivodeships from 4.9 (Warmińsko-mazurskie) to 7.4 (Zachodnio-pomorskie).

The study group consisted of 852 children with isolated LRDs. The upper limbs were affected more often (615 children, or 72.2%) than the lower limbs (223 children, or 26.2%). Both upper and lower LRDs (1.4%) were affected in 12 children. In 2 cases (0.2%), the affected limb was unknown.

Table 2 summarizes the comparisons of isolated LRDs with the control group. Among the most significant risk factors associated with isolated LRDs were low birth weight (≤2499 g), prematurity (≤36 months), and maternal smoking history.

Lower birth weight (≤2499), prematurity (≤36 months), and maternal smoking history are strongly associated with isolated LRDs. Low birth weight under 2499 g was associated with a 2-fold increase in the risk of isolated LRDs (OR (odds ratio) [95% CI (confidence interval)] = 2.41 [1.66, 3.48]), and birth weight above 4000 g was associated with a nearly two-fold decrease in risk (0.52 [0.36, 0.74]). These associations were robust enough to adjust for other significant risk factors (adjusted OR [95% CI] = 2.38 [1.54, 3.66] and adjusted OR [95% CI] = 0.40 [0.25, 0.63], respectively).

In addition, gestational age <36 weeks was significantly associated with isolated LRDs, increasing the risk by nearly 50% (OR [95% CI] = 1.47 [1.04, 2.09]), and this association also remained statistically significant after adjustment for known risk factors (adjusted OR [95% CI] = 1.62 [1.06, 2.48]).

Even more striking was the association with mothers who had a history of smoking during the first three months of pregnancy. This factor was significantly associated with isolated LRDs, increasing the risk by nearly 4-fold, both before (OR [95%CI] = 4.30 [2.88, 6.42]) and after adjustment for all other risk factors (adjusted OR [95%CI] = 4.18 [2.77, 6.30]).

In addition to the strong associations above, several interesting trends also emerged. For example, there was a clear trend for a lower level of education among parents of children with LRDs. Interestingly, a strong trend was observed for the lowest paternal education level: paternal primary and basic education was associated with over two-fold increase in the risk of isolated LRDs (OR [95%CI] = 2.57 [1.67, 3.95]) and OR [95%CI] = 2.06 [1.55, 2.74], respectively). The risk of LRDs was also greatest in mothers with only primary and basic occupational education (OR [95%CI] = 1.64 [1.17, 2.29] and OR [95%CI] = 1.38 [1.03, 1.83], respectively). Notably, after adjustments for other risk factors, these associations remained statistically significant only for the level of paternal education.

Table 3 presents the analysis of specific pregnancy exposures that were tested for association with isolated LRDs, including chronic and acute maternal conditions during pregnancy.

Most notably, maternal infections of the upper respiratory tract occurring in the first three months of pregnancy were strongly associated with a 3- to 4-fold increased risk of isolated LRDs, both before and after adjustment for other risk factors (OR [95%CI] = 4.29 [2.34, 7.84] and adjusted OR [95%CI] = 3.04 [1.59, 5.79], respectively). Consistent with this association, we detected a significant trend for increased risk with exposure to anti-inflammatory drugs; exposures to these drugs in the first trimester were associated with 5.5-fold increased risk in our models. Antibiotic exposure was significant in univariate analyses, but the fully adjusted model provided only suggestive evidence for this risk factor, not meeting our significance threshold (adjusted OR [95%CI] = 2.44 [1.00, 5.95]).

The second strong association was detected for gestational hypertension. Gestational hypertension was significantly more common among pregnancies with isolated LRDs compared to healthy controls; the risk of LRDs was increased by over three-fold (OR [95%CI] = 3.69 [1.76, 7.77]). Even after correcting for all other risk factors, this association remained statistically significant (adjusted OR [95%CI] = 3.81 [1.44, 10.07]).

Lastly, we performed geo-temporal analysis of the LRDs across the area and time period covered by the PRCM. Using Kulldorff’s scan, we did not recognize statistically significant spatial (*p* = 0.8100), or spatiotemporal clusters (*p* = 0.3380).

## 4. Discussion

The overall prevalence of LRDs in the years 1998–2010 in the Polish population (5.9 per 10,000 births) was similar to that of the EUROCAT (5.8 per 10,000 births) [26]. However, a detailed analysis of regions covered by the PRCM shows a large degree of regional variation, ranging from 4.9 to 7.4 per 10,000 births (Table 1). Although a similar overall prevalence of LRD was observed in the Alberta Congenital Anomalies Surveillance System (ACASS) across Canada (5.6 per 10,000 births) [18]; additional prevalence comparisons with countries that are not part of the EUROCAT reporting system are difficult because of the lack of reporting standardization.

The analysis of the temporal trend in the prevalence in the Wielkopolska region (the region that meets the Full Member criteria of EUROCAT [14]) over the period of 11 years (1998–2008) showed a stable rate of LRDs in contrast with a decreasing trend reported for other European countries [15].

Due to the above observations, an attempt was made to identify possible clusters of LRDs in the Polish population. Clusters of other congenital birth defects such as gastroschisis and cleft lip and palate in the PRWWR area have previously been identified [27,28]. Nevertheless, our Kulldorff’s scan for geo-temporal clusters of the LRDs across the area and time period covered by the PRCM did not identify any statistically significant clusters.

LRDs constitute a heterogeneous group of limb defects with variable clinical manifestations and diverse etiologies. Recent progress in molecular biology and genetics enabled the discovery of the molecular basis of selected types of LRDs [29,30]. However, the etiology of the majority of LRDs remains unknown, and several studies highlighted the potential involvement of environmental factors [2,3,4,5,11,12,31,32].

The considerable sample size of LRDs cases that were reported to the PRCM along with the large and well-characterized group of healthy control births allowed for carrying out well-powered epidemiological analyses of these rare defects.

Our data demonstrate that lower birth weight and prematurity are associated with isolated LRDs. The inverse correlation between birth weight and risk of LRDs has been demonstrated previously [33,34,35]. It is difficult to explain why LRDs would be associated with shortened gestation or lower birth weight, and common risk factors cannot be excluded. It is well established that there is a correlation between low birth weight and many environmental and socio-economic factors, i.e., unemployment, lower occupational status, or single mother status [36,37].

Our data point to an increased risk of LRDs when there is a lower level of paternal education. Parental education represents a marker of socio-economic status that is also inversely correlated with many health behaviors, including smoking and drug use [24,38].

Importantly, the risk of LRDs and history of maternal smoking were also appreciably associated, and we demonstrated an over three-fold higher risk of having a baby with LRD among mothers with a smoking history during the first trimester of pregnancy. These observations are consistent with several prior studies [31,32,39,40].

Several potential mechanisms have been discussed. One is an increased risk of chronic fetal hypoxia [41]. It has been suggested that vascular damage caused by smoking or drugs during organogenesis may represent one of the potential mechanisms [31].

Another hypothesis is that elevated homocysteine levels found in smokers [42] interfere with the conversion of retinol to retinoic acid [43]. Retinoic acid controls molecular signal pathways of limb differentiation [44].

Some epidemiological studies suggested that genetic susceptibilities contribute to limb reduction defects in combination with maternal smoking during pregnancy. For instance, *NAT1*, *NAT2*, *GSTT1*, *GSTM1*, and *NOS3* genetic variants increased the risk for limb reduction defects in infants whose mothers were active smokers during pregnancy [45].

We detected no significant relationships between parental age and the risk of LRDs. Similar to our study, the lack of association between maternal age and isolated LRDs was also found in the Medical Birth Registry of Norway [46].

Lastly, in our analysis, pregnancy complications were significantly more common among pregnancies with isolated LRDs. The effects of chronic medical conditions and complications in mothers of children with LRDs have previously been studied. For example, it has been recognized that there is a greater frequency of LRDs in infants of diabetic mothers [4,46,47,48]. Conversely, the EUROCAT study did not confirm the association of LRDs with pre-gestation maternal diabetes [49]. Unfortunately, in this study we only had a single case with pre-existing diabetes (type I); thus, we were not able to test this potential risk factor.

We observed an association between maternal gestational hypertension and the risk of LRDs, but the pathogenic link between the two remains unknown. Maternal hypertension after fetal organogenesis is unlikely to represent a risk factor for birth defects. It is thus more likely that there are common risk factors for fetal malformation and gestational hypertension [50]. The existence of several common risk factors, such as obesity, are well documented. Among mothers of children with LRDs in our analysis, only one reported pre-existing hypertension, but we cannot rule out that pregnant women with hypertension throughout pregnancy had this condition unrecognized prior to conception. Moreover, maternal hypertension has been identified as a risk factor for several other birth defects, such as esophageal atresia and stenosis [51], or hypospadias [52,53]. The most likely mechanism could be related to compromised uteroplacental perfusion [52]. The WHO’s (World Health Organization) international survey showed that newborns of women with chronic maternal hypertension have a four-fold increase in the risk of limb malformations [54]. However, it should be noted that the WHO survey took into account all limb defects, not only LRDs, which limits the comparison with our results.

Acute infections and exposure to anti-inflammatory drugs in the first three months of pregnancy also represented significant risk factors. Our analyses indicated that upper respiratory tract infections in early pregnancy were associated with a heightened risk of isolated LRDs. Associations between LRDs and the common cold or flu with fevers in the first trimester of pregnancy have been reported previously [55]. Fever and cytokine storm may constitute a teratogenic factor. In fact, the teratogenic effects of fevers have been reported for other birth defects, e.g., neutral tube defects and cleft lip and palate [56]. It is also possible that the fever is a marker of a more severe infection.

An important limitation of our work is that we are missing information on the types of infections, occurrence and severity of fevers, or specific treatments used for infection. Thus, based on the available information from PRCM, it is not possible for us to further examine the associations between the risk of LRDs and prenatal infections or related medication use. Moreover, due to relatively small case numbers and missing data, we are unable to examine interactions between infections and exposure to anti-inflammatory agents. Future work will be needed to address this hypothesis.

There are three additional important limitations to our study. First, both live births and stillbirths are monitored; however, only live births are recorded with multiple source surveillance systems that offer the completeness of data on congenital malformations. The data for stillbirths is incomplete. Given this limitation, our analysis only considers cases of live birth.

Second, in the geospatial analysis, we rely on reporting forms with data on maternal residence during pregnancy. However, maternal mobility during pregnancy was not captured by the reporting forms. We feel that this limitation is relatively minor because Poles are characterized by a relatively minor level of mobility. Approximately 65% of Poles spend their entire adult life in one place, and of those who move, the vast majority (67%) relocates within the same voivodeship, usually no more than 50 km from their previous place of residence [57].

Third, our study is also limited by registry data, which has the intrinsic property of possibly being susceptible to misdiagnoses. For this reason, all reported cases have been extensively reviewed by a clinical geneticist to recognize and exclude those that do not meet our stringent inclusion requirements. All cases with suggestive genetic etiology were excluded. According to suggestions from the literature, amniotic band syndromes were also excluded from our analysis [58]. However, not all children with isolated limb defects were examined in genetic clinics, which could cause misclassification in a small number of cases. For instance, it is possible that some types of syndromes with LRDs may not be recognized and thus may be misclassified as isolated defects. For instance, the only clinical manifestation of Holt–Oram syndrome (the result of a mutation in gene *TBX5*) might be a size reduction or malformation of a thumb [29].

## 5. Conclusions

Our study supports the hypothesis that maternal factors play an important role in the etiology of isolated LRDs. Further research is needed to define the precise mechanisms underlying our reported associations. Despite a large variation in the prevalence of LRDs in individual regions of the Polish population, no consistent geo-temporal clusters of LRDs were identified.

## Figures and Tables

**Table 1:** The number of cases and prevalence per 10,000 births of limb reduction defects (LRDs) for 11 Polish voivodeships covered by the Polish Registry of Congenital Malformations (PRCM) from 1998 to 2010.

11 Polish Voivodeships	N. of Cases of LRDs *	N. of Births *	Prevalence
Dolnośląskie ^#^	223	350,122	6.4
Kujawsko-pomorskie ^#^	154	280,888	5.5
Lubelskie ^&^	137	196,933	7.0
Lubuskie ^#^	72	135,498	5.3
Opolskie ^#^	69	116,562	5.9
Podkarpackie ^&^	113	191,537	5.9
Pomorskie ^#^	163	318,847	5.1
Śląskie ^$^	252	431,350	5.8
Warmińsko-mazurskie ^#^	108	221,049	4.9
Wielkopolskie ^#^	287	477,095	6.0
Zachodniopomorskie ^#^	162	219,120	7.4
Total	1740	2,939,001	5.9

* live births and stillbirths; ^#^ 1998–2010; ^$^ 2001–2010; ^&^ 2002–2010.

**Table 2:** The comparison of baseline characteristics between cases with isolated LRDs (*N* = 852, PRCM 1998–2010) and population-matched healthy controls. The *p*-values and odds ratios (OR) were derived based on univariate (unadjusted) and multivariate (fully adjusted) logistic regression models, as described in the methods.

Variables:	Isolated	Healthy	Unadjusted Model		Adjusted Model	
	LRDs	Controls			(Gestational Age/Birth Weight, Maternal Tobacco Use, Gravidity)	
	*N* = 852 ^b^	*N* = 852 ^b^	OR Crude [95%CI]	*p*-Value	OR Adjusted [95%CI]	*p*-Value
Gestational age (GA) in weeks:						
≤36	80 (9.4%)	59 (6.9%)	1.47 [1.04, 2.09]	0.0313	1.62 [1.06, 2.48]	0.0266
37–41	705 (82.7%)	765 (89.8%)	reference		reference	
≥42	33 (3.9%)	28 (3.3%)	1.28 [0.76, 2.14]	0.3482	1.23 [0.68, 2.22]	0.4915
Birth weight (BW) in grams:						
≤2499	102 (12.0%)	44 (5.2%)	2.41 [1.66, 3.48]	<0.0001	2.38 [1.54, 3.66]	0.0001
2500–3999	684 (80.3%)	710 (83.3%)	reference		reference	
≥4000	49 (5.8%)	98 (11.5%)	0.52 [0.36, 0.74]	0.0003	0.40 [0.25, 0.63]	0.0001
Sex:						
Male	479 (56.2%)	441 (51.8%)	1.21 [1.00, 1.46]	0.0507	1.25 [0.99, 1.57]	0.0586
Female	369 (43.3%)	411 (48.2%)	reference		reference	
Place of residence:						
Rural	337 (39.6%)	355 (41.7%)	reference		reference	
Urban	508 (59.6%)	497 (58.3%)	1.08 [0.89, 1.31]	0.4544	0.98 [0.78, 1.24]	0.8612
Maternal age in yeras:						
≤24	293 (34.4%)	289 (33.9%)	1.06 [0.85, 1.33]	0.6055	0.96 [0.73, 1.26]	0.7632
25–29	296 (34.7%)	310 (36.4%)	reference		reference	
30–34	152 (17.8%)	176 (20.7%)	0.90 [0.69, 1.18]	0.4648	0.89 [0.64, 1.23]	0.4842
≥35	95 (11.2%)	77 (9.0%)	1.29 [0.92, 1.82]	0.1397	0.95 [0.61, 1.47]	0.8176
Paternal age in yeras:						
≤24	147 (17.3%)	128 (15.0%)	1.25 [0.94, 1.67]	0.1217	1.26 [0.89, 1.79]	0.1913
25–29	283 (33.2%)	309 (36.3%)	reference		reference	
30–34	207 (24.3%)	230 (27.0%)	0.98 [0.77, 1.26]	0.8900	1.08 [0.80, 1.46]	0.6093
35–39	101 (11.9%)	95 (11.2%)	1.16 [0.84, 1.60]	0.3658	1.03 [0.68, 1.54]	0.8970
≥40	64 (7.5%)	58 (6.8%)	1.20 [0.82, 1.78]	0.3493	1.12 [0.69, 1.82]	0.6547
Maternal education:						
Primary	129 (15.1%)	108 (12.7%)	1.64 [1.17, 2.29]	0.0039	0.98 [0.65, 1.48]	0.9369
Basic occupational	218 (25.6%)	217 (25.5%)	1.38 [1.03, 1.83]	0.0284	0.97 [0.69, 1.36]	0.8686
High school	298 (35.0%)	331 (38.8%)	1.23 [0.95, 1.61]	0.1219	0.95 [0.69, 1.29]	0.7289
Higher	143 (16.8%)	196 (23.0%)	reference		reference	
Paternal education:						
Primary	74 (8.7%)	50 (5.9%)	2.57 [1.67, 3.95]	<0.0001	1.87 [1.11, 3.15]	0.0185
Basic occupational	348 (40.8%)	293 (34.4%)	2.06 [1.55, 2.74]	<0.0001	1.53 [1.10, 2.14]	0.0123
High school	223 (26.2%)	283 (33.2%)	1.37 [1.01, 1.84]	0.0400	1.15 [0.81, 1.62]	0.4432
Higher	105 (12.3%)	182 (21.4%)	reference		reference	
Gravidity:						
1	407 (47.8%)	401 (47.1%)	reference		reference	
2	226 (26.5%)	261 (30.6%)	0.85 [0.68, 1.07]	0.1669	0.95 [0.73, 1.23]	0.6877
≥3	202 (23.7%)	150 (17.6%)	1.33 [1.03, 1.71]	0.0280	1.21 [0.90, 1.62]	0.2069
Previous miscarriages ^a^						
No	318 (74.3%)	307 (74.7%)	reference		reference	
Yes	104 (24.3%)	96 (23.4%)	1.05 [0.76, 1.44]	0.7827	0.84 [0.57, 1.25]	0.3960
Previous terminations of pregnancy ^a^						
No	418 (97.7%)	396 (96.4%)	reference		reference	
Yes	2 (0.5%)	4 (1%)	0.47 [0.09, 2.60]	0.3898	0.25 [0.02, 2.45]	0.2316
Previous stillbirths ^a^						
No	400 (93.5%)	386 (93.9%)	reference		reference	
Yes	7 (1.6%)	4 (1.0%)	1.69 [0.49, 5.81]	0.4062	1.36 [0.27, 6.70]	0.7092
Maternal tobacco use						
No	491 (57.6%)	718 (84.3%)	reference		reference	
Yes	103 (12.1%)	35 (4.1%)	4.30 [2.88, 6.42]	<0.0001	4.18 [2.77, 6.30]	<0.0001

^a^ 2nd pregnancy and subsequent; ^b^ marginal totals for some variables may be different because of missing values; Odds ratio (OR), confidence interval (CI), birthweight (BW), gestational age (GA), limb reduction defects (LRDs).

**Table 3:** Specific exposures during pregnancy and their associations with the risk of isolated LRDs.

	Isolated LRDs	Controls	Unadjusted Model		Fully Adjusted Model (Gestational Age/Birth Weight, Maternal Tobacco Use, Gravidity)	
			OR Crude [95%CI]	*p*-Value	OR Adjusted [95%CI]	*p*-Value
Chronic maternal diseases						
Thyroid diseases	8 (3.1%)	8 (2.3%)	1.37 [0.51, 3.69]	0.5364	1.45 [0.46, 4.63]	0.5274
Pre-existing diabetes mellitus	1 (0.4%)	7 (2.0%)	0.20 [0.02, 1.60]	0.1279	0.38 [0.04, 3.45]	0.3898
Asthma	5 (2.0%)	10 (2.8%)	0.68 [0.23, 2.03]	0.4930	0.25 [0.05, 1.24]	0.0900
Pre-existing hypertension	1 (0.4%)	2 (0.6%)	1.46 [0.13, 16.21]	0.7570	0.78 [0.07, 8.73]	0.8430
Epilepsy	5 (2.0%)	4 (1.2%)	1.71 [0.45, 6.43]	0.4274	0.53 [0.05, 5.10]	0.5792
Acute complications during pregnancy						
Infection of upper respiratory tract (1st trimester)	47 (15.8%)	15 (4.2%)	4.29 [2.34, 7.84]	<0.0001	3.04 [1.59, 5.79]	0.0007
Infection of urinary tract (1st trimester)	9 (3.5%)	11 (3.1%)	1.12 [0.46, 2.74]	0.8053	1.07 [0.42, 2.75]	0.8865
Infections of the genital tract (1st trimester)	2 (0.8%)	9 (2.6%)	0.30 [0.07, 1.42]	0.1299	0.36 [0.08, 1.67]	0.1904
Gestational hypertension	27 (9.7%)	10 (2.8%)	3.69 [1.76, 7.77]	0.0006	3.81 [1.44, 10.07]	0.0071
Gestational diabetes mellitus	6 (2.3%)	13 (3.7%)	0.63 [0.24, 1.68]	0.3582	0.30 [0.06, 1.47]	0.1387
Oligohydramnios	11 (4.2%)	7 (2.0%)	2.15 [0.82, 5.62]	0.1188	2.91 [0.96, 8.79]	0.0588
Polyhydramnios	6 (2.3%)	2 (0.6%)	4.10 [0.82, 20.50]	0.0854	3.85 [0.72, 20.69]	0.1163
Medications in the 1st trimester of pregnancy						
Antibiotics	19 (7.1%)	9 (2.6%)	2.89 [1.29, 6.49]	0.0103	2.44 [1.00, 5.95]	0.0502
Anti-inflammatory drugs	12 (4.6%)	3 (0.9%)	5.47 [1.53, 19.59]	0.0090	5.58 [1.51, 20.67]	0.0101
Antiepileptic drugs	4 (1.6%)	3 (0.9%)	1.82 [0.40, 8.22]	0.4340	1.64 [0.26, 10.51]	0.6032

## Data Availability

The data presented in this study are available on request from the corresponding author. The data are not publicly available due to the need to obtain approval from the PRCM team for using the data.
